# Feasibility of peripheral OCT imaging using a novel integrated SLO ultra-widefield imaging swept-source OCT device

**DOI:** 10.1007/s10792-021-01837-7

**Published:** 2021-04-08

**Authors:** Simrat K. Sodhi, John Golding, Carmelina Trimboli, Netan Choudhry

**Affiliations:** 1 University of Cambridge, Cambridge, UK; 2 Vitreous Retina Macula Specialists of Toronto, 3280 Bloor St. West. Suite 310, Etobicoke, ON M8X 2X3 Canada; 3 Department of Ophthalmology and Visual Sciences, University of Toronto, Toronto, ON Canada; 4 Cleveland Clinic Canada, Toronto, ON Canada

**Keywords:** Peripheral, Optical coherence tomography, Mid-periphery, Far periphery, Full-field, Swept-source OCT

## Abstract

**Purpose:**

To describe the feasibility of peripheral OCT imaging in retinal diseases using a novel full-field device.

**Methods:**

A total of 134 consecutive eyes were referred and imaged on the Optos Silverstone swept-source OCT (SS-OCT) (Optos PLC; Dunfermline, UK). Scanning laser ophthalmoscope (SLO) images and the associated SS-OCT images were obtained in the posterior pole, mid-periphery or far periphery based on the nature of the referral and on new areas of interest observed in the optomap images at the time of imaging.

**Results:**

A total of 134 eyes (96 patients) were enrolled in the study. One hundred and twenty-five eyes (91 patients) with 38 retinal pathologies were prospectively assessed and 9 eyes (5 patients) were excluded due to incomplete image acquisition. The average age of the subjects was 54 years (range 21–92 years). Thirty-nine out of 125 eyes (31%) had macular pathologies. Eighty-six out of 125 eyes (69%) had peripheral only pathologies, an area which cannot be visualized by standard OCT devices with a 50 degree field-of-view.

**Conclusions:**

The ability to capture peripheral pathologies using an integrated SLO-UWF imaging with full-field swept-source provided high-grade anatomical insight that confirmed the medical and surgical management in a majority of cases. Its use in the mid- and far periphery provides a holistic clinical picture, which can potentially aid in the understanding of various retinal pathologies.

**Supplementary Information:**

The online version contains supplementary material available at 10.1007/s10792-021-01837-7.

## Introduction

Since its advent in 1991, optical coherence tomography (OCT) imaging has revolutionized diagnostics in ophthalmology and the understanding of normal, benign and pathologic macular features [1]. The noninvasive nature combined with its high-resolution structural imaging has made it an indispensable tool in both retina practices and investigative clinical trials [2, 3]. However, its use in peripheral retinal imaging is infrequent thereby bypassing the site of many vision-threatening pathologies, including retinal tears, holes, detachments and diagnostic masqueraders, such as peripheral retinoschisis [4]. To date, clinical examination has been the gold-standard for detecting such pathologies independent of ultra-widefield (UWF) and OCT imaging.

Few studies have utilized spectral-domain OCT (SD-OCT) to image the far retinal periphery using standard widefield (50 degree) field-of-view (FOV) systems [5]. This is primarily due to the limitation of standard OCT devices ability to access the mid- and far-peripheral retina with a centrally fixed laser head. A steerable laser head available on devices such as the Heidelberg Spectralis (Heidelberg Engineering, Heidelberg, Germany) have been successfully used to image the peripheral retina [5]. Despite a steerable laser head, there are potential pitfalls which arise, including: optical distortions, limited depth range, increased scan time for multiple scan positions, patient compliance and post-processing time if images are montaged [6].

UWF devices capture the retina beyond the anterior border of the vortex veins (in all four quadrants) in a single image and have increased sensitivity and specificity in recognizing retinal pathologies compared to standard two-field imaging [7]. UWF systems combined with previous modalities, including fluorescein angiography (FA), indocyanine green angiography (ICGA), pseudocolor and fundus autofluorescence (FAF), have enhanced our understanding of the peripheral retina [8]. OCT was subsequently added to peripheral imaging; however, these iterations involved steering-based image acquisition or montage techniques. Advances in OCT technology have led to the development of swept-source OCT (SS-OCT), a system that incorporates a 1050 nm wavelength laser and an axial scan rate of 100,000 A scans/sec to provide penetration to visualize deeper ocular structures and faster scan-speeds, resulting in shorter acquisition times [7]. Until now, SS-OCT was limited to widefield (WF) systems, limiting the perspective to include the retina up to the posterior border of the vortex veins in all quadrants. Expanding the FOV to an UWF perspective allows imaging to the pars plana, resulting in high-resolution, cross-sectional scans of the periphery. Imaging of the peripheral vitreous, retina and choroid addresses the dearth of high-quality imaging of the retinal periphery and may enhance clinical decision making related to a variety of vitreo-retinal and choroidal disorders.

The purpose of this study was to evaluate the feasibility of high-quality, SS-OCT images of the retinal periphery using a novel scanning laser ophthalmoscope (SLO)-UWF imaging device with integrated full-field OCT capabilities (Optos Silverstone; Optos PLC; Dunfermline, UK). In this context, full-field refers to the ability to perform traditional OCT of the posterior pole as well as beyond the vortex veins encompassing both the mid-periphery and far periphery. Furthermore, our aim was to determine the feasibility of said imaging in a real-world clinical practice. Compared to previous peripheral imaging reports, we assessed whether this novel device can acquire high-quality, peripheral retinal images without the need for montage or steering.

## Methods

Selection of participants.

In this prospective, single-center, observational case series, patients referred for both peripheral and unknown pathologies were consecutively enrolled and analyzed at the Vitreous Retina Macula Specialists of Toronto, between February and July 2020. A total of 96 patients (134 eyes) were initially enrolled in the study. After review, 5 patients were removed due to incomplete imaging, leaving 91 patients (125 eyes). Patients were consecutively chosen based on the inclusion criteria and could have one or both eyes enrolled in the study. Inclusion criteria included: (i) age 18 or older, (ii) ability to provide informed consent, (iii) referral for central or peripheral retina pathology and (iv) attempt made to image peripheral pathology. This study was approved by a centralized Institutional Review Board and followed the tenets of the Declaration of Helsinki. Written informed consent was obtained from all participants and was in accordance with current ICH/GCP guidelines, Sect. 4.8.10.

Visits and imaging.

Following enrollment, patient history was collected at baseline, along with visual acuity, clinical exam including tonometry, slit-lamp examination with a Volk digital wide field lens, dilated fundus examination and peripheral SS-OCT. At each subsequent visit, a full clinical exam was performed which included tonometry, visual acuity testing and peripheral SS-OCT. Patients were treated according to the physician’s discretion.

At baseline, all patients had SS-OCT testing using the Optos Silverstone swept-source OCT. SS-OCT imaging was performed by two experienced ophthalmic photographers (J.G., C.T) using a single machine. The SS-OCT system provides a 200° single-capture optomap image. An OCT scan is then performed and registered onto the optomap image. The OCT scan is executed with a scanning speed of 100,000 A scans per second. It utilizes a wavelength-sweeping laser, with a central wavelength of 1,050 nm wavelength. At baseline, color and green AF optomap images, and UWF 6 mm line and 6 mm volume OCT scans were obtained for all patients. Based on possible pathology identified, additional scans were acquired. Typically, a UWF 6 mm HD volume and 23 mm extended line OCT scans were also captured, at the photographer’s discretion,

Following referrals, each patient’s images were assessed on several specific areas of interest by a single physician, which included confirmation of diagnosis, pathology location and ability to capture peripheral pathology. If a patient had multiple pathologies, each pathology was analyzed separately. Pathology location was classified as posterior pole, mid-periphery or far periphery based on the classifications and guidelines for wide field imaging from the International Wide Field Imaging Study Group [9]. As previously published, the posterior pole defined as the area of retina within the major temporal vascular arcades and slightly just beyond; mid-periphery is the region of retina extending from the vascular arcades to the posterior edge of the vortex vein ampulla and far periphery as the region of retina anterior to the vortex vein ampulla.

## Results

A total of 91 patients (125 eyes) assessed in this study. The average age of the subjects was 54 years (range 21–92 years). Fifty-three of the 91 patients were female and 38 were male. There were no serious adverse events throughout the course of the study.

A total of 134 eyes (96 patients) were enrolled in the study. A totla of 125 eyes (91 patients) were assessed and 9 eyes (5 patients) were excluded due to incomplete image acquisition. Eighty-six out of 125 eyes (69%) had peripheral only pathologies. Frequent peripheral pathologies included: retinal tears (11 eyes), retinal holes (10 eyes), retinoschisis (10 eyes), retinal detachment (RD) (10 eyes), of which 5 eyes were assessed post-pneumatic retinopexy (PnR), retinal tuft (7 eyes), central serous retinopathy (CSR) (5 eyes), lattice degeneration (4 eyes) and choroidal nevus (4 eyes). Three of the eyes (2.4%) had pathologies that were not accessible by the full-field SS-OCT device. These included a retinal tear, retinal hole and a status post-PnR. In 39 out of 125 eyes (31%), the pathology was located in the posterior pole, and while the images were acquired, these pathologies would have been captured by standard OCT devices with a 50 degree field of view (Table 1).

**Table 1:** A summary of the feasibility of peripheral OCT for various surgical and medical pathologies present in the posterior pole, mid-periphery or far periphery

Pathology (*n*, eyes)	Affected eye (OS, OD, OU)	Pathology location (1 = posterior pole; 2 = mid-periphery; 3 = far periphery)	Ability to capture peripheral pathology (Y/N)
Retinal tear (*n* = 11)	OS	2	Y
	OD	3	Y
	OS	2	Y
	OU	3	Y
	OD	3	N
	OS	3	Y
	OD	3	Y
	OU	3	Y
	OD	2, 3	Y
Retinal hole (*n* = 10)	OD	2	Y
	OS	3	Y
	OD	3	Y
	OD	3	Y
	OS	2	Y
	OS	2	Y
	OU	3	N
	OD	2	Y
	OS	3	Y
Retinoschisis (*n* = 10)	OS	3	Y
	OU	3	Y
	OS	1, 2	Y
	OU	3	Y
	OD	1	Y
	OS	1	Y
	OU	3	Y
Retinal tuft (*n* = 7)	OD	2, 3	Y
	OS	1	Y
	OS	2	Y
	OD	3	Y
	OD	3	Y
	OS	3	Y
	OD	2	Y
Retinal detachment (RD) (*n* = 5)	OS	1, 2, 3	Y
	OS	1, 2, 3	Y
	OS	2, 3	Y
	OU	1	Y
s/p Pneumatic retinopexy (PnR) (*n* = 5)	OS	2	N
	OS	2	Y
	OU	3	Y
	OS	1, 2	Y
Central serous retinopathy (CSR) (*n* = 5)	OD	2	Y
	OS	1	Y
	OU	1	Y
	OS	1	Y
Lattice degeneration (*n* = 4)	OS	2	Y
	OD	2, 3	Y
	OD	3	Y
	OS	3	Y
Choroidal nevus (*n* = 4)	OD	2	Y
	OS	2	Y
	OD	1,2	Y
	OS	1	Y
Chorioretinal atrophy (*n* = 4)	OU	3	Y
	OD	1	Y
	OD	2	Y
RVO (*n* = 4)	OS	1	Y
	OU	1	Y
	OD	1	Y
Diabetic retinopathy (DR) (*n* = 4)	OU	1	Y
	OU	1	Y
Retinitis pigmentosa (RP) (*n* = 4)	OU	2	Y
	OU	2	Y
Dry age-related macular degeneration (dARMD) (*n* = 4)	OU	1	Y
	OU	1	Y
Congenital retinal pigment epithelial hypertrophy (CHRPE) (*n* = 3)	OS	2	Y
	OS	3	Y
	OD	3	Y
Posterior vitreous detachment (PVD) (*n* = 3)	OD	1	Y
	OU	1	Y
White without pressure (WWOP) (*n* = 3)	OD	3	Y
	OU	2	Y
Optic pit maculopathy (*n* = 2)	OD	3	Y
	OD	1	Y
Floaters (*n* = 2)	OU	1	Y
Myopic degeneration (*n* = 2)	OU	1	Y
Vitreous adhesion (*n* = 2)	OU	2	Y
Adult vitelliform dystrophy (*n* = 2)	OU	1	Y
Retinal thinning (*n* = 2)	OU	2	Y
Unknown peripheral streaks (*n* = 2)	OU	2	Y
Punctate inner choroidopathy (PIC) (*n* = 2)	OU	1, 2	Y
Vogt–Koyanagi–Harada (VKH) disease (*n* = 2)	OU	2	Y
Cone dystrophy (*n* = 2)	OU	1	Y
Dark without pressure (DWP) (*n* = 2)	OU	3	Y
Uveitis (*n* = 2)	OU	2	Y
Multifocal choroiditis (*n* = 2)	OU	2, 3	Y
Choroidal lesion (*n* = 1)	OD	1	Y
Myelinated nerve fiber layer (NFL) ( *n* = 1)	OS	2	Y
Linear peripheral drusen (*n* = 1)	OD	3	Y
Peripheral drusenoid pigment epithelium detachment (PED) (*n* = 1)	OD	2	Y
Vitreous condensation (*n* = 1)	OS	2	Y
Geographic atrophy (GA) (*n* = 1)	OS	1	Y
Macular pucker (*n* = 1)	OS	1	Y
Choroidal melanoma (*n* = 1)	OS	2	Y
S/p open globe repair (OGR) (*n* = 1)	OS	2	Y

## Discussion and summary

This is the first report of peripheral OCT imaging using a novel full-field swept-source OCT device (Optos Silverstone; Optos PLC). Previous studies captured peripheral imaging using spectral-domain OCT (SD-OCT) or SS-OCT with a wide FOV, but to our knowledge, this is the first iteration of UWF SS-OCT peripheral imaging [5, 6, 10–13].

Based on the 38 pathologies captured and assessed in this study, peripheral OCT was most impactful in surgical cases such as retinal tears, holes and detachments. These surgical pathologies can be diagnosed without the use of peripheral OCT imaging, but analysis of the anatomical features these peripheral images provided confirmed if surgical intervention was necessary. These pathologies are seldom imaged with in-office OCT devices prior to surgical procedures, contributing to an omission of vital anatomical features. Steering-based and intraoperative OCT (iOCT) devices can be used to capture peripheral images; however, the former reduces the resolution and is technically challenging, while the latter is scarcely available [14]. Traditionally, operating strategies rely on the location of tear, historical outcomes and techniques used in training or adopted from published papers. With OCT-based approaches, the extent of retinal tears, holes and detachments can be visualized, subclinical holes or tears can be uncovered, and recovery can be tracked.

**Fig. 1:**
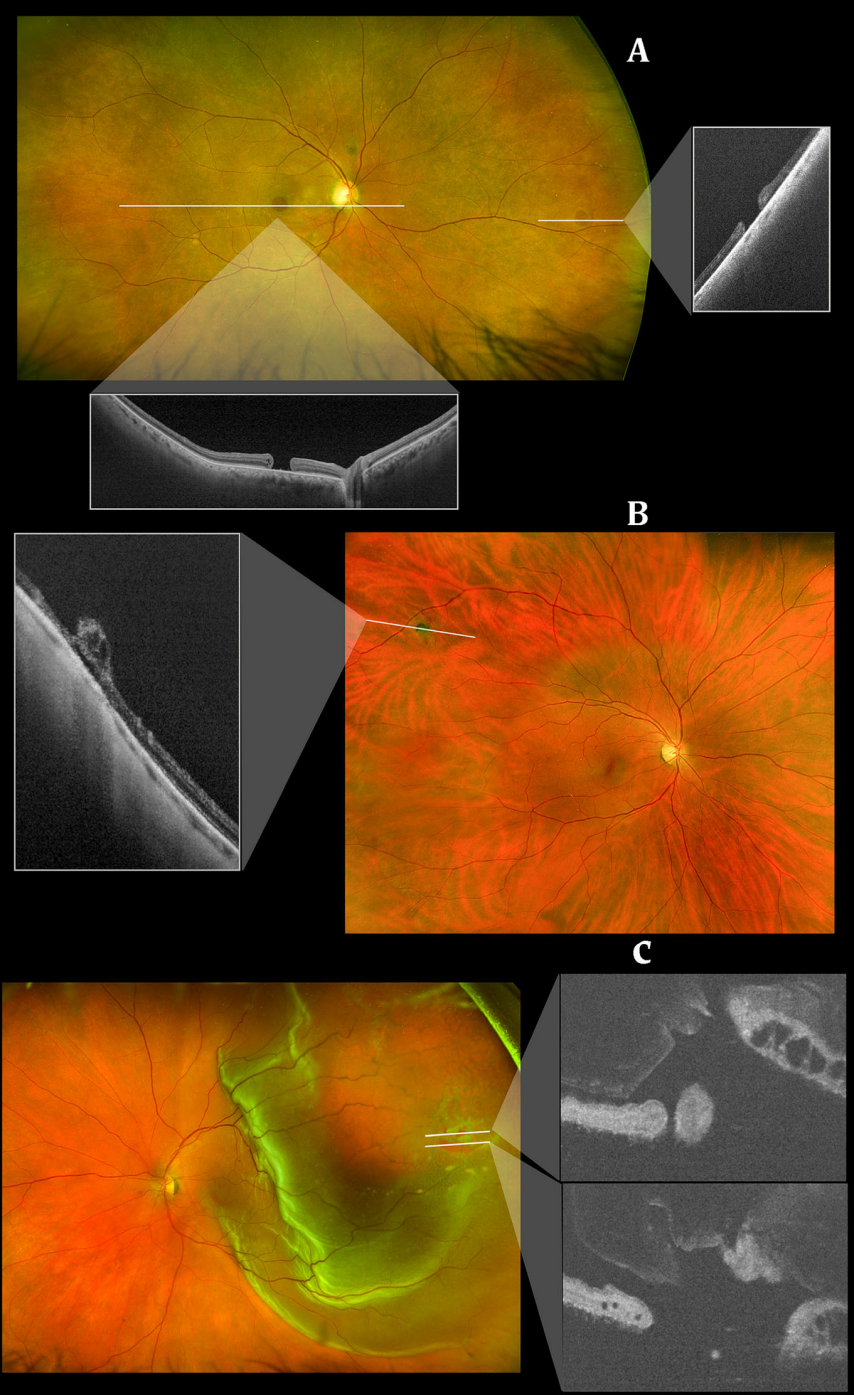
Ultra-widefield (UWF) color scanning laser ophthalmoscope (SLO) images and corresponding insets depicting cross-sectional swept *source* OCT scans of: **a** A macular hole and atrophic retinal hole in the far periphery **b** cystic retinal tuft in far periphery and **c** retinal detachment extending from posterior pole to far periphery with retinal tear in mid-periphery

Capturing peripheral pathologies can also assist in the differentiation of lesions previously misidentified. Retinal tufts (Fig. 1B) are can be commonly mistaken for retinal holes and themselves difficult to differentiate from the cystic and non-cystic variety. The risk of a cystic retinal tuft leading to retinal detachment is 1%, and thus, prophylactic treatment of cystic retinal tufts is not advised [15]. However, without confirmation of the pathology, premature treatment may be initiated. In Fig. 1B, a cystic retinal tuft in the far periphery would have been missed with traditional macular imaging. After imaging, it allowed the physician to laser the retinal tuft amidst an active posterior vitreous detachment, for which the patient had symptoms of flashing lights and floaters. Conversely, lattice degeneration, due to thinning of the peripheral retinal tissue, is more vulnerable to developing tears, breaks, or holes. It is causally related to retinal detachments, especially in young myopes, which would potentially benefit from laser retinopexy. Retinal holes are often present in myopes and have been estimated to occur in approximately 2–5% of the population [16]. Byer observed a 43% incidence of round atrophic holes in patients with lattice degeneration of the retina [17]. In 2% of eyes, clinical or progressive subclinical retinal detachment occurred. In the presence of subretinal fluid, if confirmed, these patients benefit from laser photocoagulation, thereby reducing the risk of retinal detachment. Figure 1a depicts a macular hole, but a secondary retinal hole is also present in the far periphery. Peripheral imaging allowed visualization of both holes and led to pars plana vitrectomy for the macular hole, while the retinal hole was confirmed as atrophic and monitored. Figure 1c exhibits a retinal detachment extending from the posterior pole to far periphery with a retinal tear present in the mid-periphery. The peripheral retinal imaging shows the extent of the detachment as well as the presence of a tear and was used with serial OCTs to monitor PnR response postoperatively.

Choroidal lesions, such as choroidal nevi, traditionally require fluorescein angiography (FA) to provide information on the fluid status. In a study of 120 patients, Shields et al. evaluated choroidal nevi utilizing time domain (TD) OCT and determined that numerous overlying changes such as subretinal fluid, retinal edema, retinal thinning and photoreceptor attenuation are visible by OCT [18]. Peripheral imaging of these lesions will thus forgo the need for invasive FA testing. Choroidal nevi have been reported to transform into melanomas in 2%, 9% and 13% of eyes at 1, 5 and 10 years, respectively [19]. Early detection of melanomas when the malignancy is small is critical in improving patients’ survival, thus underlining the importance of early imaging and subsequent monitoring of progression.

**Fig. 2:**
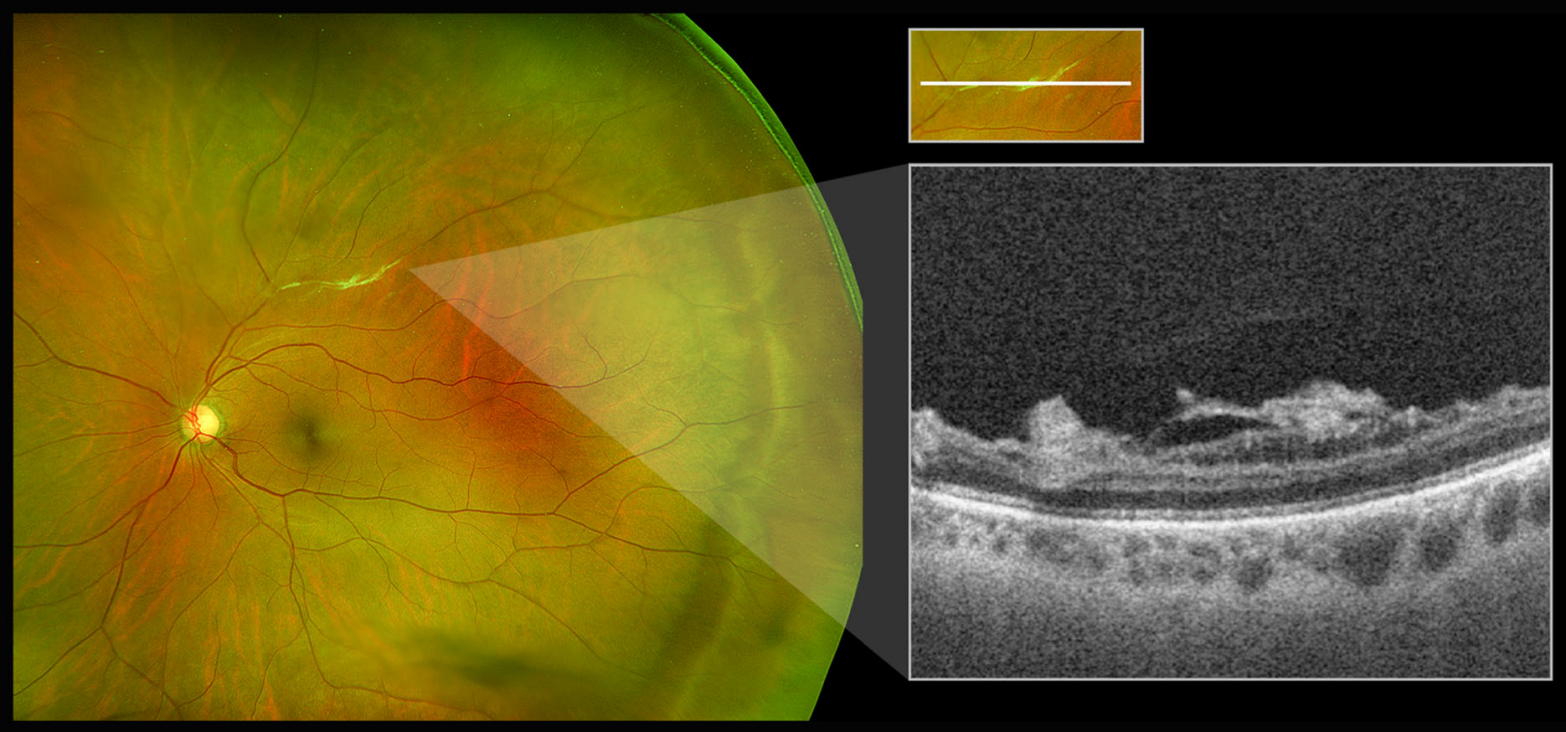
Ultra-widefield color scanning laser ophthalmoscope image and magnified inset of vitreous condensation in mid-periphery

Primary eye-care referrals for unknown pathologies are encountered frequently and require stringent imaging to capture faint alterations. In instances where there is a macular issue, this device does not add any additional benefit as the pathology would have been captured in a standard 50-degree view by most machines. However, in differentiating common peripheral pathologies such as white without pressure (WWOP) and dark without pressure (DWP), which are routinely referred by primary eye-care clinics, the addition of peripheral OCT can allow for image capture and potential diagnosis in the hands of a primary eye-care physician, while offering anatomical confirmation by a retina specialist. Another example is observed in Fig. 2, where one of the superior blood vessels in the mid-periphery has a pale, yellow appearance. Without mid-peripheral OCT, this may be diagnosed as vascular sheathing or a sclerotic vessel secondary to retinal vascular disease, but cross-sectional SS-OCT reveals this abnormality as vitreous condensation.

**Fig. 3:**
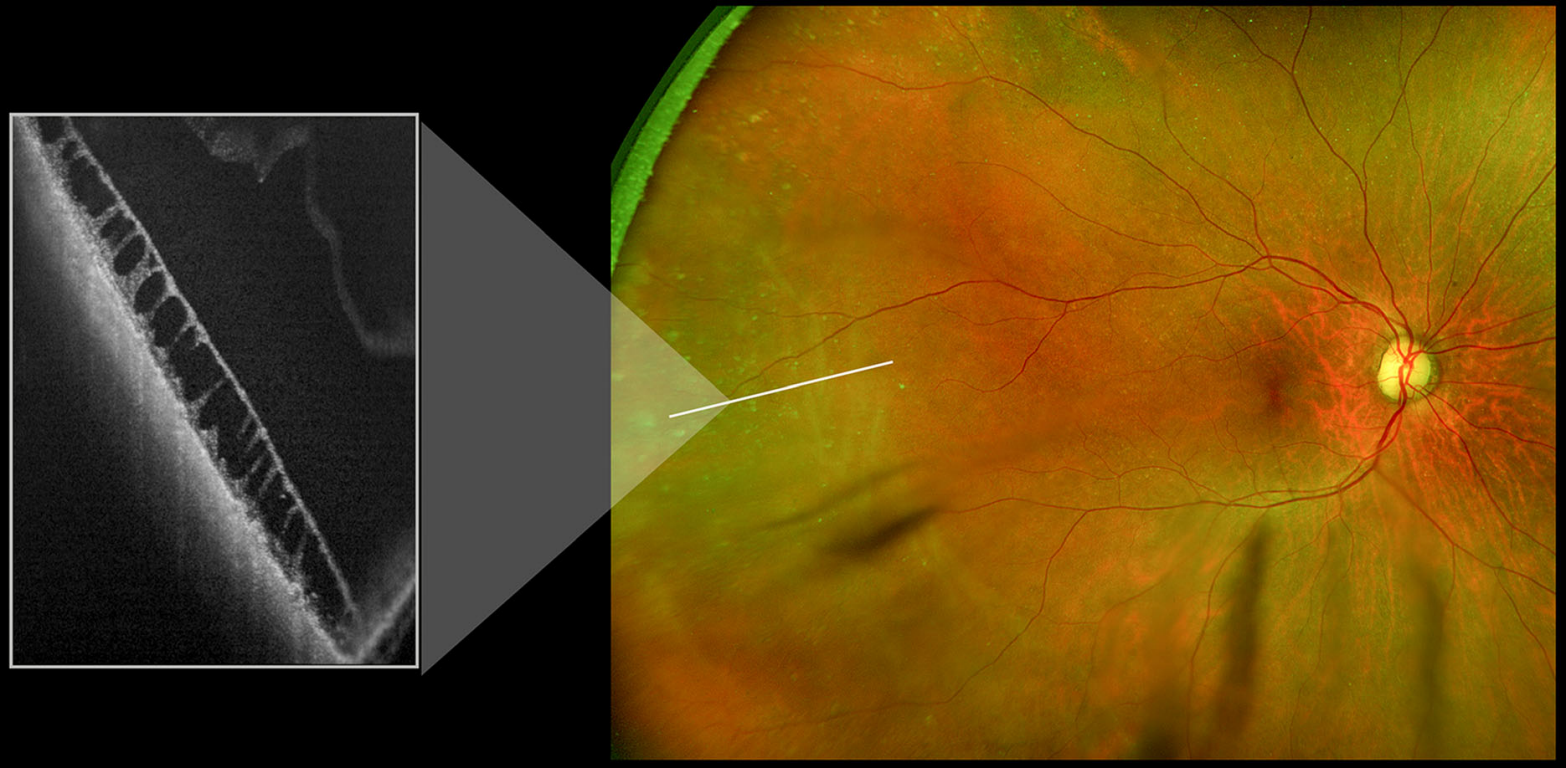
Ultra-widefield color scanning laser ophthalmoscope image and magnified inset of retinoschisis in far periphery

In our study, there were several instances where subtle peripheral pathologies were present and the ophthalmic photographer independently captured them without physician guidance. There was a standard operating procedure in place for image acquisition, but certain cases required the addition of isolated scan types to completely capture the pathologies. These scenarios demonstrate the importance of working with an attentive and astute photographer, as you can rely on their knowledge for correct image acquisition. In Fig. 3 for example, the posterior pole and mid-periphery appeared relatively normal on clinical exam; however, a subclinical senile retinoschisis was detected in the far periphery. This schisis was not visible on any of the routine UWF pseudocolor or FAF imaging.

One of the limitations of this full-field combination UWF and SS-OCT device is that it only allows OCT capture when the fixation light is central. If the gaze is required to be moved either superior, inferior, temporal or nasal, the fixation light would also move and no longer allow for an OCT image capture. In order to bypass this discrepancy, the ophthalmic photographer confined the fixation light to the center, while manually guiding patient’s gaze. Without this technique, UWF line scans would only be available for pathologies visible in the primary gaze. This hindrance may be resolved in future iterations of the software, but required an innovative procedure in this study.

The device itself forgoes steering or composite imaging and instead associates the OCT to an already registered scanning laser ophthalmoscopy (SLO) image. This prevents scanning and refocusing the device at each new area, thereby making clinical correlation and operator use easier. During image acquisition, patients are not required to tilt their field of gaze, which increases the chances of procuring a precise image and eases the strain felt by patients. As previously mentioned, swept source utilizes a higher wavelength and scan speed, which increases the resolution while decreasing acquisition time. Presently, the resolution is limited when attempting detailed imaging of the vitreous. However, vitreous adhesion is visible in cases of retinal tears (Video 1).

There was a perceived improvement in the clinical management of patients, but this is hard to quantify as it depends on multiple factors, including (i) the imaging protocol used in the clinic, (ii) the skill level of the photographer capturing the images, (iii) the specialty of the physician analyzing the images (retina specialist versus doctor of optometry) and (iv) the other imaging modalities available to the physician (rural versus urban setting). The ability to capture OCT scans of peripheral pathologies without the need to montage or steer demonstrates the feasibility of this full-field swept-source OCT device. However, our study was not powered to determine the clinical utility of this device. A case–control study, which compares the results of a traditional clinical exam to this OCT and to traditional OCT models, that have been reported to image the peripheral retina, would be necessary to comment on the clinical utility of this device. We found the use of this device useful in confirming surgical pathologies, but future studies would need to enroll more patients from a wider range of surgical pathologies to determine if this device impacted the clinical management of those cases.

As demonstrated in this study, peripheral retinal imaging provides fundamental anatomical details, such as fluid status, that have the potential to aid in the management of retinal cases. There are multiple instances where a full-field SS-OCT device would be advantageous, but three are particularly important:

(I) Surgical cases, where OCT is not traditionally adopted, subtle features provide insight into strategies that can yield optimal outcomes, such as retinal detachment and monitoring the effectiveness of intervention (i.e., pneumatic retinopexy).
(II) Referrals for ‘unknown’ pathologies or unexplained symptoms that rely on a wider scope of imaging to discern marginal changes or when differentiating between clinical masqueraders, such as retinal detachment and retinoschisis.
(III) Telemedicine initiatives where precise diagnoses and management of these entities are obligated to occur in after-hours, remote or resource-poor settings where retinal specialists may not be as readily available.

In summary, this novel, UWF, SS-OCT peripheral imaging approach provides high-quality anatomical information that allowed confirmation of diagnoses, especially in surgical cases. Montage or steering techniques to reach the periphery could have been used, but our approach forgoes the need for these techniques, thus increasing the speed and resolution of imaging while providing patient facileness. In a retinal practice, this approach offers a novel addition to the ophthalmic clinical exam thereby providing comprehensive options to routine clinical imaging for virtually all pathologies.

## Supplementary Information

Below is the link to the electronic supplementary material. Swept source OCT volume scan video depicting vitreous adhesion to a retinal tear with cystic retinal changes.

Supplementary file1 (MP4 2191 kb)

## Data Availability

The datasets generated during and/or analyzed during the current study are available from the corresponding author on reasonable request.
